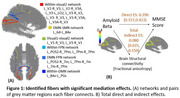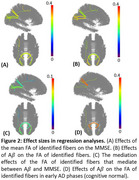# Mapping Structural Connectome Patterns in Alzheimer's Disease

**DOI:** 10.1002/alz70855_104137

**Published:** 2025-12-24

**Authors:** Tengfei Li, Xifeng Wang, Martin Cole, Zhengwu Zhang, Hongtu Zhu

**Affiliations:** ^1^ University of North Carolina at Chapel Hill, Chapel Hill, NC, USA; ^2^ University of Rochester, Rochester, NY, USA

## Abstract

**Background:**

We performed fibertracking tractography based on the ADNI dataset to examine how brain structural connectivity mediates Aβ's impact on cognitive decline in Alzheimer's Disease (AD).

**Method:**

We performed fibertracking tractography and fiber clustering on the ADNI2 dataset using Tractoflow and TractDL pipelines to extract central fibers within each fiber bundle. Mean fractional anisotropy (FA) was computed per fiber per visit. The Cerebrospinal fluid (CSF) Aβ42, Mini Mental State Examination (MMSE) scores, and MRI data were matched by visits. Linear mixed models tested Aβ→FA and FA→MMSE associations (Bonferroni corrected). Age, age², sex, age‐by‐sex interaction, education, and total brain volume were controlled as confounders. Fibers linked to both Aβ and MMSE underwent FDR‐adjusted mediation analyses. Identified Aβ–FA associations were further explored in cognitively normal (CN) subjects as early‐phase AD effects.

**Result:**

Data includes 287 visits from 205 subjects (53 AD, 132 mild cognitive impairment, and 102 CN). 16 fibers were identified with both Aβ→FA and FA→MMSE associations: nine from within‐visual networks, one within‐default mode network (DMN), two within‐frontoparietal (FPN), three DMN‐FPN, and one DMN‐sensorimotor (SMN), all positively mediating between the Aβ and MMSE. Aβ was positively associated with FA for the six DMN or FPN‐related fibers during early AD phases. Aβ's direct and total indirect effects were 0.290 ([0.157, 0.423]) and 0.080 ([0.025, 0.150), respectively.

**Conclusion:**

Aβ's impact on cognitive decline was partly mediated (21.6%) by FA changes—primarily within visual, DMN, FPN, and DMN‐FPN networks. DMN and FPN connectivity appears especially susceptible in early AD phases.